# Inhibition of the histone demethylase, KDM5B, directly induces re-expression of tumor suppressor protein HEXIM1 in cancer cells

**DOI:** 10.1186/s13058-019-1228-7

**Published:** 2019-12-05

**Authors:** Monica M. Montano, I-Ju Yeh, Yinghua Chen, Chris Hernandez, Janna G. Kiselar, Maria de la Fuente, Adriane M. Lawes, Marvin T. Nieman, Philip D. Kiser, James Jacobberger, Agata A. Exner, Matthew C. Lawes

**Affiliations:** 1 Department of Pharmacology, Case Western Reserve University School of Medicine, 10900 Euclid Avenue, Cleveland, OH 44106 USA; 2 Department of Physiology and Biophysics, Case Western Reserve University School of Medicine, 10900 Euclid Avenue, Cleveland, OH 44106 USA; 3 General Medical Sciences, Case Western Reserve University School of Medicine, 10900 Euclid Avenue, Cleveland, OH 44106 USA; 4 Department of Radiology, and Center for Proteomics and Bioinformatics, Case Western Reserve University School of Medicine, 10900 Euclid Avenue, Cleveland, OH 44106 USA; 5 Oncostatyx, 11000 Cedar Avenue Suite 26, Cleveland, OH 44106 USA

**Keywords:** HEXIM1 inducers, KDM5B, Breast cancer, Differentiation

## Abstract

**Background:**

The tumor suppressor actions of hexamethylene bis-acetamide (HMBA)-inducible protein 1 (HEXIM1) in the breast, prostate, melanomas, and AML have been reported by our group and others. Increased HEXIM1 expression caused differentiation and inhibited proliferation and metastasis of cancer cells. Historically, HEXIM1 has been experimentally induced with the hybrid polar compound HMBA, but HMBA is a poor clinical candidate due to lack of a known target, poor pharmacological properties, and unfavorable ADMETox characteristics. Thus, HEXIM1 induction is an intriguing therapeutic approach to cancer treatment, but requires better chemical tools than HMBA.

**Methods:**

We identified and verified KDM5B as a target of HEXIM1 inducers using a chemical proteomics approach, biotin–NeutrAvidin pull-down assays, surface plasmon resonance, and molecular docking. The regulation of HEXIM1 by KDM5B and KDM5B inhibitors was assessed using chromatin immunoprecipitation assays, RT-PCR, western blotting, and depletion of KDM5B with shRNAs. The regulation of breast cancer cell phenotype by KDM5B inhibitors was assessed using western blots, differentiation assays, proliferation assays, and a mouse model of breast cancer metastasis. The relative role of HEXIM1 in the action of KDM5B inhibitors was determined by depleting HEXIM1 using shRNAs followed by western blots, differentiation assays, and proliferation assays.

**Results:**

We have identified a highly druggable target, KDM5B, which is inhibited by small molecule inducers of HEXIM1. RNAi knockdown of KDM5B induced HEXIM1 expression, thus validating the specific negative regulation of tumor suppressor HEXIM1 by the H3K4me3/2 demethylase KDM5B. Known inhibitors of KDM5B were also able to induce HEXIM1 expression, inhibit cell proliferation, induce differentiation, potentiate sensitivity to cancer chemotherapy, and inhibit breast tumor metastasis.

**Conclusion:**

HMBA and 4a1 induce HEXIM1 expression by inhibiting KDM5B. Upregulation of HEXIM1 expression levels plays a critical role in the inhibition of proliferation of breast cancer cells using KDM5B inhibitors. Based on the novel molecular scaffolds that we identified which more potently induced HEXIM1 expression and data in support that KDM5B is a target of these compounds, we have opened up new lead discovery and optimization directions.

## Background

Hexamethylene bis-acetamide (HMBA) is a small molecule that has been investigated due to its notable anti-cancer and cell differentiation activities [1, 2]. However, HMBA failed in phase II clinical trials because of a toxicity, thrombocytopenia, low potency, and short half-life requiring infusion at a high dosage [1, 3]. HMBA induces terminal differentiation via upregulation of HMBA-inducible protein 1 (HEXIM1), albeit at mM levels [4]. HEXIM1 also plays a central role in the anti-cancer activities of another category of therapeutics, BET inhibitors [5].

We have been studying the role of HEXIM1 as a tumor suppressor whose expression is lost during breast and prostate tumor progression and metastasis [6–8]. Moreover, our findings were corroborated by a Cancer Outlier Profile Analysis (COPA) of existing microarray data, showing HEXIM1 is downregulated and a potential tumor suppressor in breast, prostate, and other cancers [9]. HEXIM1 expression was inversely related to proliferative activity in breast tumor tissue [6]. The anti-cancer actions of HEXIM1 in breast and prostate cancer, melanomas, and AML have been reported by our group and others [5, 10, 11].

To develop analogs of HMBA as improved chemical tools and pharmaceutical leads, we previously synthesized symmetrical and unsymmetrical derivatives of HMBA [12]. Since the direct target of HMBA was unknown at that time, the derivatives were generated via a traditional medicinal chemistry strategy, i.e., ligand-based modification. One of the compounds, 4a1, exhibited enhanced potency when compared to HMBA in prostate cells [12] and breast cancer cells [13], and inhibited metastasis.

To determine the mechanism by which HMBA and 4a1 upregulate HEXIM1 expression and to develop even more potent HEXIM1 inducing compounds, we utilized 4a1 as a bait in a chemical proteomics approach to identify direct binding targets. We determined that 4a1 binds to KDM5B and that the ensuing inhibition of KDM5B activity resulted in upregulation of active histone marks H3K4me2 on the *HEXIM1* gene and induction of HEXIM1 expression. Our data also suggest that upregulation of HEXIM1 expression levels plays a critical role in the inhibition of proliferation, differentiation, and regulation of expression of major growth regulatory factors in breast cancer cells by KDM5B inhibitors.

## Methods

### Biotin–NeutrAvidin pull-down assay

Extracts from MDA-MB-231 cells were utilized in biotin–NeutrAvidin pull-down assays and as described in detail in Additional file 1. The resulting gel was visualized with coomassie blue staining for mass spectrometry.

### Mass spectrometry

Bands visualized by coomassie blue staining were in-gel digested using trypsin. LC-MS analyses were performed as described previously [14] and in detail in Additional file 1.

### Purification of KDM5B JmjC domain

KDM5B cDNA cloned into pFB-LIC-Bse (from Structural Genomics Consortium, University of Oxford, UK) was expressed in Sf9 cells as previously described [15]. The protein purification is described in detail in Additional file 1. The purified KDM5B Jmj domain was used in surface plasmon resonance studies.

### Surface plasmon resonance

SPR studies were performed using a Biacore T100 (GE Healthcare, USA) and described in detail in Additional file 1.

### Docking of HEXIM1 inducers onto KDM5B

Coordinates for the KDM5B-KDOAM25 complex were retrieved from the PDB (accession code 5A3N). Coordinate files for 4A1 and hexamethylene-bis-acetamide (HMBA) were generated using the GRADE server and converted to .pdbqt format using Autodock tools. Further details on docking are provided in Additional file 1.

### Cell culture, transfections, and lentiviral infection

MCF7 and TNBC lines were obtained from the American Tissue Culture Collection in April 2017 and were maintained based on the instructions from ATCC. KDM5B shRNA and HEXIM1 shRNA lentiviruses were generated as described in Additional file 1. Breast cancer cells were transduced with lentiviruses for 12–16 h. TNBC cells were harvested 36 h after infection with lentiviruses. Puromycin was used to select for cells expressing shRNAs. Cells were transfected with control or expression vector for FLAG-KDM5B using FuGENE HD (Promega) according to the manufacturer’s instructions.

### Chromatin immunoprecipitation

Cells were processed for ChIP analyses as described previously [6] and described in more detail in Additional file 1.

### RT-PCR

Total mRNAs were extracted and processed for RT-PCR analyses as described in more detail in Additional file 1.

### Western blotting

Cell lysates were analyzed by Western blotting as described previously [16] and described in more detail (including antibodies utilized) in Additional file 1.

### Lipid droplets (Nile red staining)

Cells were stained with Nile red (marker of cell differentiation) as described previously [13] and described in more detail in Additional file 1.

### Proliferation assay

Cell proliferation was assessed using the MTT based Cell Growth Determination Kit (Sigma-Aldrich) and described in more detail in Additional file 1.

### Colony formation assay

Five hundred cells were aliquoted onto 12-well plates and fed fresh growth media (with DMSO or KDOAM25) every 3–4 days. After 2 weeks of culture, cells were fixed with methanol at room temperature for 20 min and stained with methylene blue, and colonies were photographed.

### Flow cytometry

Cell cycle analysis was implemented with PI staining and analyses via a flow cytometry. Briefly, the cells were fixed with 70% ethanol and stained with a solution containing 50 μg/ml propidium iodide (Sigma) and 1 mg/ml RNase (Sigma) overnight at 4 °C. The stained cells were analyzed with an Attune NxT Flow Cytometer (Life Technologies, Carlsbad, CA).

### In vivo studies

All animal work reported herein have been approved by the CWRU Institutional Animal Care and Use Committee and in accordance with the National Institutes of Health guide for the care and use of laboratory animals. The maintenance and genotyping of Polyoma Middle-T antigen (PyMT) transgenic mice (obtained from Jackson Laboratories) and production of PLGA ± KDOAM25 were described previously [7]. After the appearance of palpable mammary tumors of 100 mm^3^ size, 50 μl of PL = GA or PLGA-KDOAM25 (50 μM) was injected into tumors every other week. In vivo studies are described in more detail in Additional file 1.

### Statistical analyses

Statistical significance was determined using Student’s *t* test comparison. For some comparisons probability values for the observed differences between groups were based on one-way ANOVA. A probability (*p*) value of < 0.05 was accepted as an appropriate level of significance.

## Results

### HEXIM1 expression is decreased in breast cancer and can be correlated with relapse-free survival

**Fig. 1:**
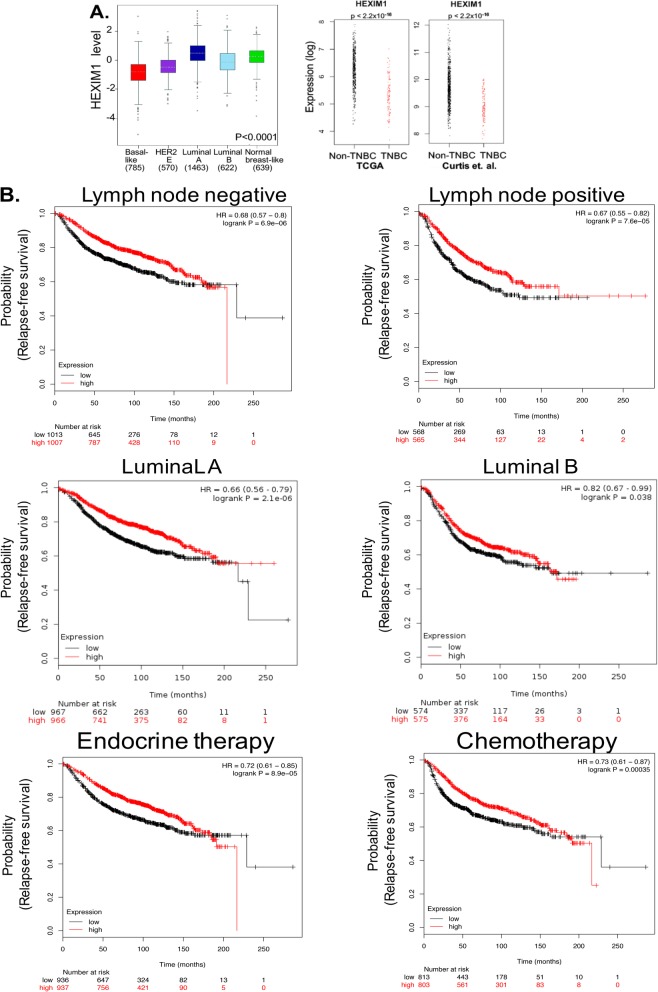
Expression of HEXIM1 in breast cancer subtypes and correlation with relapse-free survival. **a** Left panel: analyses of HEXIM1 expression in breast cancer subtypes using Breast Cancer Gene-Expression Miner Version 4 (bc-GenExMiner v.4). Right panel: analyses of HEXIM1 expression in TNBC using the TCGA and another dataset [17]. **b** Correlation of *HEXIM1* expression (probe ID 202815_s_at) in breast tumor datasets (with at least 1000 patients) with probability of relapse-free survival (RFS) as determined using the Kaplan–Meier (KM) plotter

While we have reported on the expression of HEXIM1 in different grades of breast and prostate cancer [6–8], we have not reported on expression in different breast cancer subtypes. Breast Cancer Gene-Expression Miner Version 4 (bc-GenExMiner v. 4), a database of published annotated genomic data including 5609 breast cancer patients, was used to examine HEXIM1 expression in breast cancer subtypes. While HEXIM1 is expressed at somewhat higher levels less aggressive slower growing subtypes such as normal-like and luminal breast cancer, HEXIM1 levels are notably decreased in the faster growing subtypes such as HER2 enriched and basal/TNBC breast cancer (Fig. 1a). Analyses of the TCGA and another dataset [17] indicate decreased expression of HEXIM1 in TNBC (Fig. 1a).

The Kaplan–Meier (KM) plotter database was used to correlate mRNA levels of *HEXIM1* to patient outcomes. Probability of relapse-free survival (RFS) was significantly greater with high HEXIM1 levels when analyses were not restricted to any specific subtype (data not shown). When subtypes or cohorts were examined individually, those that had at least 1000 patients (lymph node negative or positive, luminal A, luminal B, and grade 2 cases, patients following either endocrine therapy or chemotherapy) showed significantly greater RFS with higher HEXIM1 expression (Fig. 1b). HEXIM1 expression was previously inversely correlated with proliferative activity, as assessed via the expression of the prognostic marker Ki67, in all breast tumor grades [6]. While HEXIM1 is lost in TNBC, the number of TNBC cases maybe insufficient to definitively correlate HEXIM1 expression with RFS in TNBC.

### HEXIM1 inducers bind KDM5B, a histone demethylase

HEXIM1 induction is an intriguing therapeutic approach but better chemical tools than HMBA are required. Pharmacological induction of HEXIM1 expression previously lacked any known target for direct interaction with HEXIM1 inducers, which will be critical for lead discovery and optimization. To determine the mechanistic basis for induction of HEXIM1 expression by HMBA and 4a1, we utilized a chemical proteomics approach wherein biotin-conjugated 4a1 was used as a bait for putative 4a1/HMBA targets. Our focus was on proteins that bound to the NeutrAvidin-biotin-4a1 column and which were eluted by 4a1 to a higher degree than a structurally related but inactive analog of HMBA, 3e2 [12]. Putative 4a1 binding partners were then revealed by proteomic interrogation of eluates. Analyses using the Scaffold software revealed that the histone demethylase, KDM5B, is among the proteins which exhibited the highest selectivity for 4a1 relative to 3e2. KDM5B is a member of the KDM5 family of demethylases, which remove tri- and dimethyl marks from lysine 4 on histone H3 (H3K4). The KDM5 proteins share a highly conserved domain architecture, containing a catalytic Jumonji (JmjN/JmjC) domain, a DNA-binding ARID/Bright domain, a C5HC2 zinc finger, and two or three PHD fingers [18, 19]. Each ARID family member binds to a specific DNA sequence. For example, KDM5B ARID domain recognizes a “GCACA” motif and thus negatively regulates transcription activation by demethylating promoters of a specific subset of genes. Trimethylated K4 residues (H3K4me3) are bound by the PHD domain, resulting in a bent conformation in which the PHD domain interacts with the JmjC domain [20]. KDM5 enzymes catalyze the demethylation of histones in an iron (II) and 2-oxoglutarate (2-OG)-dependent reaction [21]. Many 2-OG analogs have been generated that inhibit JmjC demethylases [22]. However, the specificity of these compounds is compromised since they often also inhibit other Fe (II)- and 2-OG-dependent enzymes, such as prolyl hydroxylases [22]. More recent studies have revealed more selective KDM5 inhibitors that favor KDM5B over the other KDM5s and the KDM4/KDM6 family members with very similar JmjC domains [23].

**Fig. 2:**
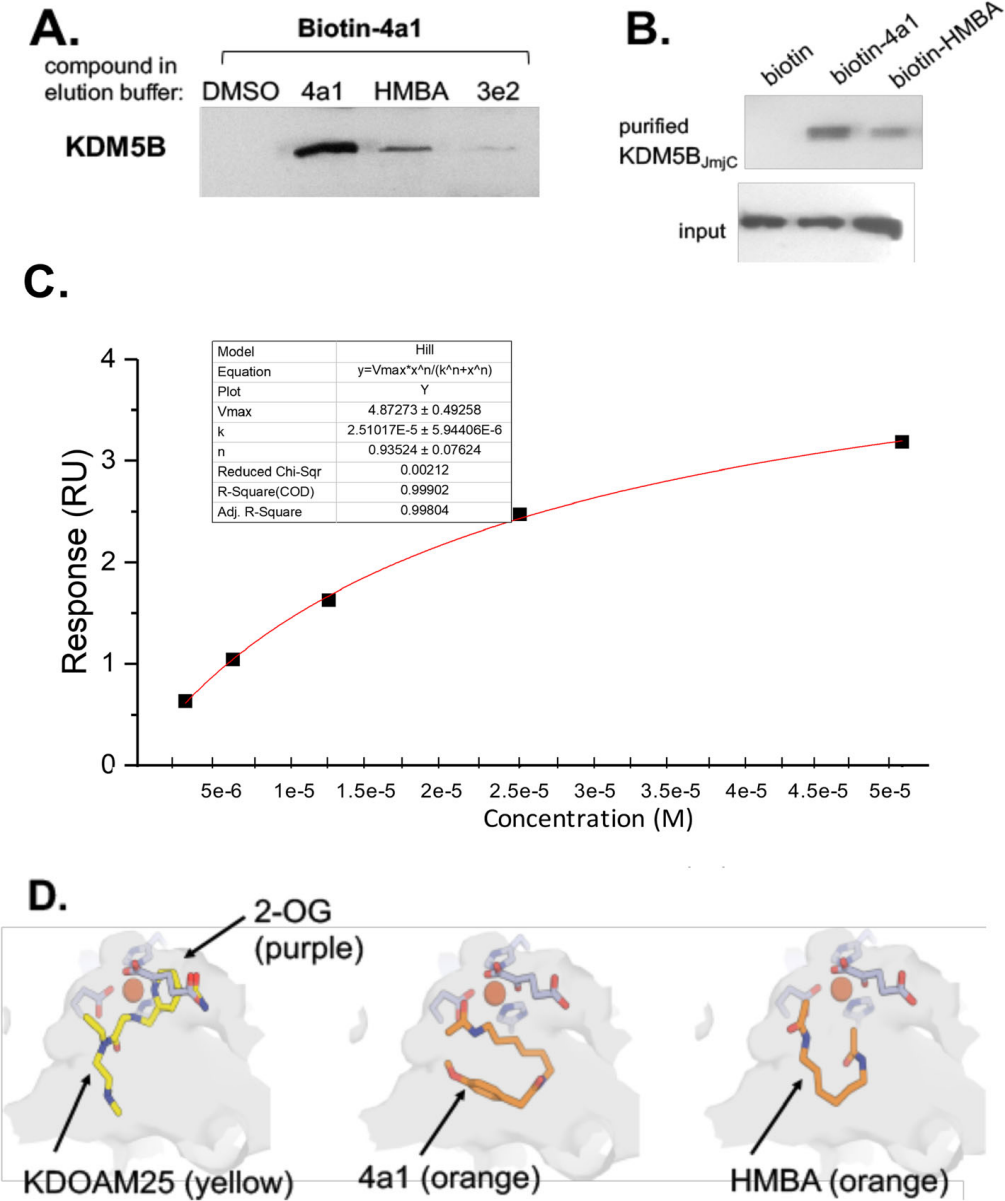
4a1 binds catalytic JmjC domain of KDM5B. **a** Biotin–streptavidin pull-down assay using NeutrAvidin beads bound to biotinylated 4a1 and whole cell lysates. Bound proteins were washed with buffer with DMSO or eluted using 4a1 (200 μM), HMBA (1 mM), or 3e2 (an inactive HMBA analog, 1 mM). Samples were subjected to SDS-PAGE and immunoblotted for KDM5B. **b** Biotin–streptavidin pull-down assay using NeutrAvidin beads bound to biotinylated 4a1 (100 μM) or biotinylated HMBA (5 mM) and purified KDM5B JmjC domain. After washes, bound proteins were eluted with SDS sample buffer and subjected to SDS-PAGE and immunoblotted for KDM5B. **c** Surface plasmon resonance analyses of the affinity of 4a1 for purified KDM5B JmjC domain. Figures are representative of at least three experiments. **d** Docking of KDOAM25, 4a1, and HMBA onto KDM5B JmjC domain

Binding of 4a1 to KDM5B was validated by incubating TNBC MDA-MB-231 lysates with biotin-conjugated 4a1. The mixture was then incubated with neutravidin resin, and KDM5B was eluted with wash buffer containing 4a1 but not eluted when wash buffer containing 3e2 was used (Fig. 2a). Binding of biotinylated 4a1 or HMBA to the catalytic JmjC domain of KDM5B is shown in Fig. 2b. Surface plasmon resonance (SPR) studies indicated a *K*_d_ value of 13 μM for 4a1 binding to JmjC domain of KDM5B (Fig. 2c).

To obtain structural insights into the binding of HMBA or 4a1 to KDM5B, we docked HMBA or 4a1 in silico against binding centers located within JmjN/JmjC catalytic domain of KDM5B based on the crystal structure PDB5A3N, which used the well-known KDM5B inhibitor KDOAM25 as ligand. Representative poses from our docking studies are shown in Fig. 2d, though a significant number of other poses with similar free energies were also obtained. Structure 5A3N suggests that KDOAM25 occupies binding sites for both 2-OG and the methylated lysine histone substrate. In contrast, our docking suggested that 4A1 and HMBA did not compete with 2-OG for its binding site but instead might occupy only the methylated lysine histone substrate binding site. 4A1 binds in a curled conformation (regardless of search parameters), while HMBA is more variable. The methoxyphenyl moiety of 4a1 was being bent around or curved and closely resembled rings if rigidified. Predicted ΔG for KDOAM25, 4a1, and HMBA bound to KDM5B are − 7.4, − 7.3, − 5.8 kcal/mol respectively.

### HEXIM1 inducers upregulated the levels of an active histone mark, H3K4me2, on the *HEXIM1* promoter

**Fig. 3:**
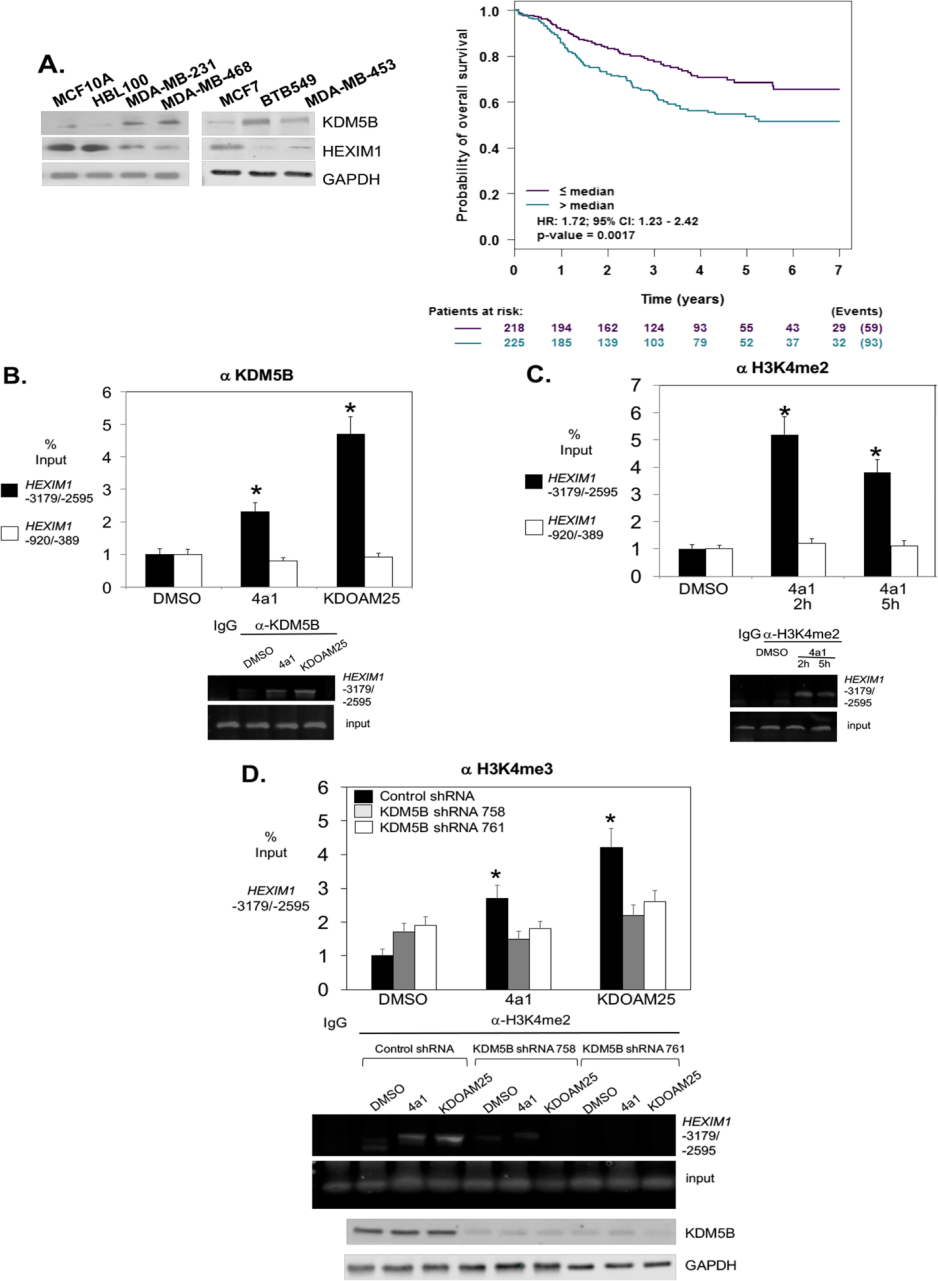
4a1 and inhibitors of the H3K4 demethylase, KDM5B, upregulated H3K4me2 levels on the HEXIM1 promoter. **a** Left panel: different breast cell lines were processed for western blot analyses of indicated proteins. Right panel: correlation of *KDM5B* expression in TNBC with probability of relapse-free survival (RFS) as determined using bc-GenExMiner v.4. **b** MDA-MB-231 cells were transfected and/or treated with DMSO, 4a1 (100 μM), or KDOAM25 (500 nM) for 2 h; ChIP assays were performed with KDM5B antibody, **c** H3K4me2 antibody, or **d** H3K4me3 antibody. PCR primers were for the indicated regions of *HEXIM1.* Representative western blots of KDM5B expression in KDM5B shRNA-transfected cells are shown in the lower panel in **d**. Figures are representative of at least three experiments. For **b** and **c**, *P < 0.01 relative to immunoprecipitated DNA from DMSO-treated cells and amplified using the same primers based on *t* test. For **d**, *P < 0.01 relative to DMSO-treated cells and transfected with the same shRNA based on *t* test

To determine the appropriate breast cell lines to utilize for testing of HEXIM1 inducers, we examined relative expression of KDM5B and HEXIM1 in cell lines representing different subtypes. Increased expression of KDM5B in TNBC cell lines and the luminal MCF7 cell line when compared to non-tumorigenic MCF10A and HBL100 (Fig. 3a) is consistent with the reported increased expression of KDM5B in breast cancer relative to normal breast tissue [24–26]. Analyses using bc-GenExMiner indicate decreased probability of overall survival with increased KDM5B expression (Fig. 3a), which has also been associated with malignancy, poor prognosis, and endocrine resistance [24–26]. Studies were conducted using TNBC cells because of our observed high expression of KDM5B in TNBC, which is consistent with other reports [27, 28] combined with the relatively low expression of HEXIM1.

We tested the possibility that HMBA and 4a1 induce HEXIM1 expression by inhibiting KDM5B, resulting in an increase in H3K4me2 marks on the *HEXIM1* regulatory region. Analysis of the *HEXIM1* 5′ regulatory region indicated two putative KDM5B ARID binding sites (GCACA) at − 3179/− 2595. Chromatin immunoprecipitation (ChIP) assays indicate KDM5B occupancy on the *HEXIM1* promoter, in particular on the 3179/− 2595 region but not on a control region (− 920/− 389) that lacks KDM5B binding sites (Fig. 3b). Of note, 4a1 did not prevent KDM5B occupancy, suggesting that 4a1 did not alter KDM5B expression, nuclear localization, or recruitment to DNA. 4a1 induced accumulation of H3K4me2 and H3K4me3 marks on the − 3179/− 2595 region but not on the control region (Fig. 3c, d). Downregulation of KDM5B using shRNA resulted in increased H3K4me2 marks on the *HEXIM1* gene (Fig. 3d). We tested whether a known inhibitor of KDM5B would increase H3K4me2 marks on the *HEXIM1* promoter. As expected, KDOAM25, a chemical tool developed by Structural Genomics Consortium, induced increased H3K4me3 marks on the *HEXIM1* regulatory region (Fig. 3d).

### Inhibitors of KDM5B histone demethylase induced HEXIM1 expression, downregulated proliferation, and upregulated differentiation of TNBC cells

**Fig. 4:**
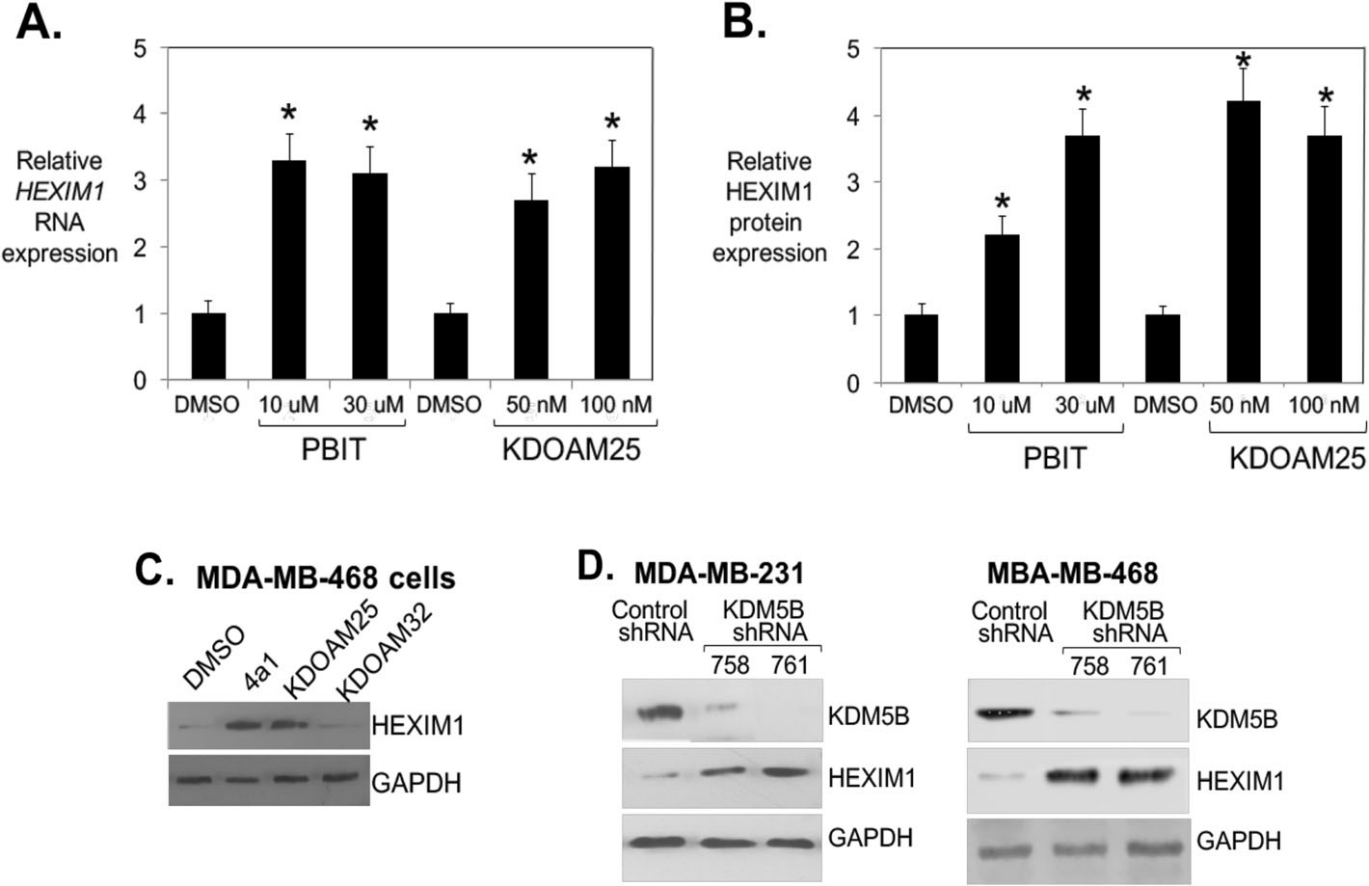
Inhibitors of the H3K4 demethylase, KDM5B, upregulated HEXIM1 expression. MDA-MB-231 cells were treated with indicated concentrations of 4a1 and KDOAM25 **a** for 5 h for RT-PCR analyses of HEXIM1 mRNA relative to GAPDH mRNA or **b** for 10 h for western blot analyses of HEXIM1 protein expression relative to GAPDH; **c** MDA-MB-468 cells were treated with 4a1 (100 μM), KDOAM25 (500 nM), or KDOAM32 (500 nM); **d** cells were processed for Western blot analyses of HEXIM1, KDM5B, or GAPDH protein expression. KDOAM32 is an inactive structurally related analog. Figures are representative of at least three experiments. For **a** and **b**, *P < 0.01 relative to DMSO-treated cells based on *t* test

Consistent with its ability to induce increases in H3K4me2 marks on the *HEXIM1* regulatory region, KDOAM25 induced significant increases in HEXIM1 expression in MDA-MB-231 and MDA-MB-468 cells (Fig. 4a–c). A known KDM5 inhibitor, 2-4(4-methylphenyl)-1,2-benzisothiazol-3(2H)-one (PBIT, Cayman Chemical, Ann Arbor, MI, ref. [29]), also increased HEXIM1 expression. In contrast, a structurally related but inactive analog of KDOAM25, KDOAM32, did not induce HEXIM1 expression (Fig. 4c). Downregulation of KDM5B expression using shRNA resulted in increased HEXIM1 expression (Fig. 4d). Thus, other KDM5 family members do not compensate for loss of KDM5B function with regard to regulation of HEXIM1 expression.

**Fig. 5:**
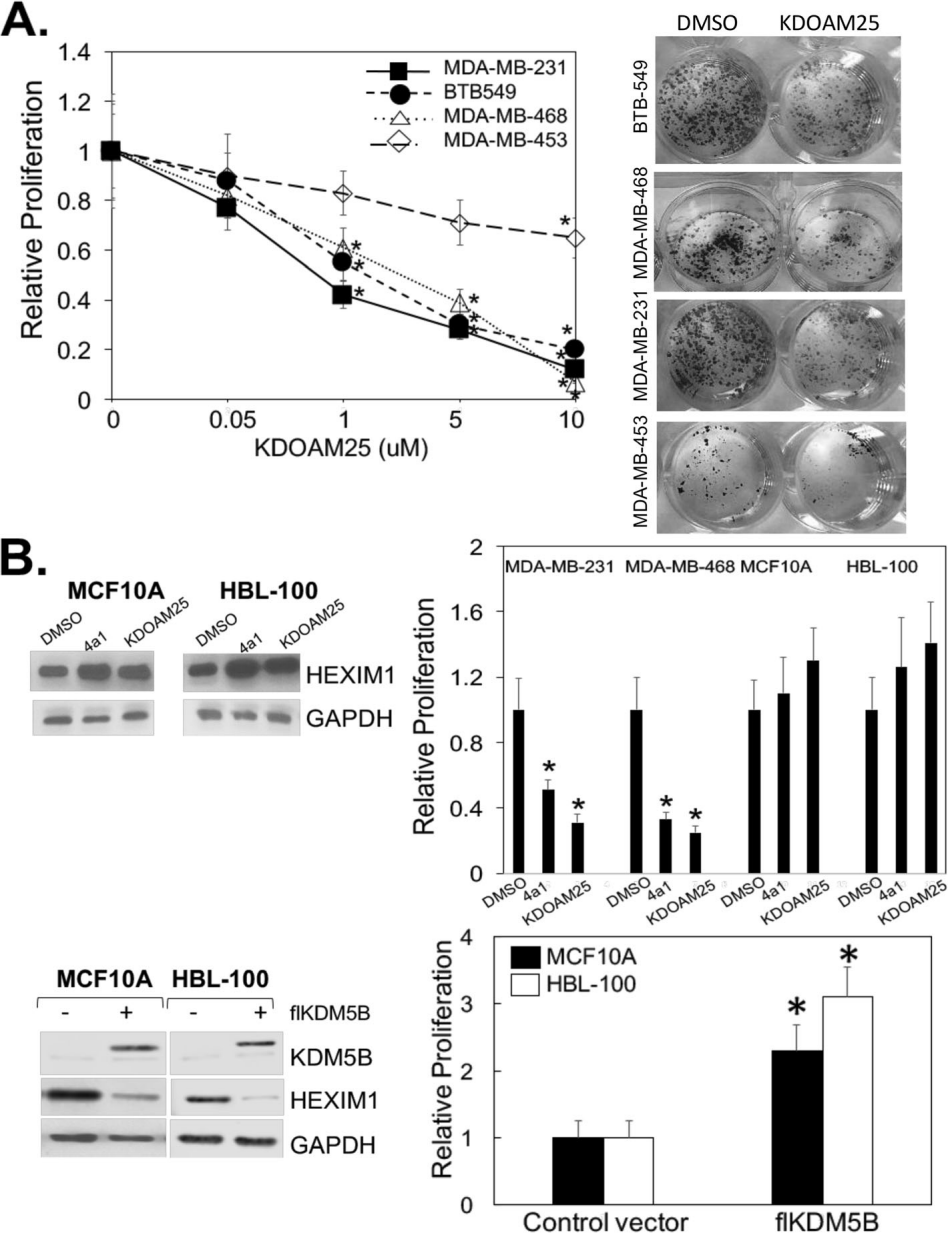
Induction of HEXIM1 expression and inhibition of proliferation of TNBC subtypes by KDM5B inhibitors. **a** Left panel: TNBC lines were treated with DMSO or indicated concentrations of KDOAM25 for 6 days and subjected to MTT assays to assess proliferation. Right panel: TNBC lines were treated with DMSO or KDOAM25 (representing IC50) for 2 weeks and subjected to colony formation assays. **b** Different breast cell lines were transfected or treated with DMSO, 4a1 (100 μM), or KDOAM25 (500 nM) for 10 h and processed for western blot analyses of indicated proteins or subjected to MTT assays to assess proliferation. Figures are representative of at least three experiments. Upper panel: *P < 0.01 relative to DMSO-treated control of the same cell line based on *t* test. Lower panel: *P < 0.01 relative to cells of the same cell line transfected with control vector based on *t* test

Studies support the role of KDM5B (JARID1B, PLU1) as an oncogene since shRNA knockdown of KDM5B inhibits proliferation in several cancer cell lines and xenograft models [25, 30, 31]. We determined whether the induction of HEXIM1 expression via KDM5B inhibitors resulted in inhibition of TNBC cell proliferation. MDA-MB-231, MDA-MB-468, BT549, and MDA-MB-453 represent basal-like (BL1) and mesenchymal stem-like (MSL), mesenchymal, and luminal androgen receptor subtypes, respectively [32]. These subtypes respond differently to a variety of targeted therapeutics [32]. KDOAM25 induced HEXIM1 expression and inhibited proliferation of various subtypes of TNBC described above, although MDA-MB-453 was less sensitive when compared to the other TNBC subtypes (Fig. 5a). Treatment with a dose of KDOAM25 corresponding to the IC_50_ resulted in decreased colony formation.

The lower expression of KDM5B non-tumorigenic cells, MCF10A and HBL100 (Fig. 3a, ref. [25, 26]), resulted in decreased sensitivity to growth inhibition with KDOAM25 when compared to TNBC cells (Fig. 5b). However, exogenous expression of KDM5B resulted in decreased expression of HEXIM1 and a corresponding increased proliferation of MCF10A and HBL100 cells.

**Fig. 6:**
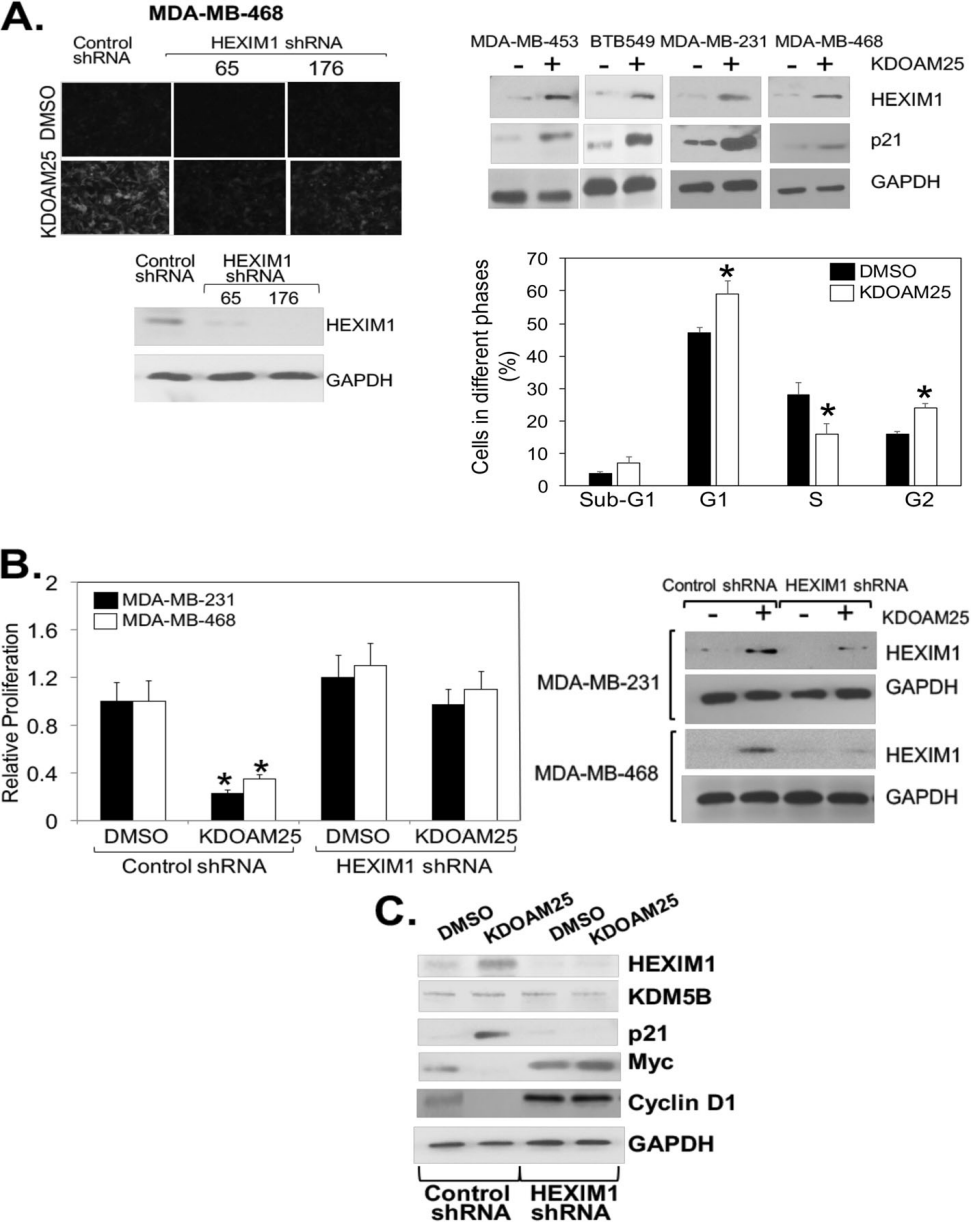
HEXIM1 is required for KDOAM25 actions. TNBC cells were transfected and/or treated with KDOAM25 (500 nM) as indicated for 10 h (for western blot analyses), 24 h (for differentiation assay and cell cycle analyses), or 6 days (for proliferation assay). **a** Cells stained with Nile Red to determine lipid droplet formation (differentiation marker, upper left panel), processed for western blot analyses of indicated proteins (upper right panel and lower left panel), or processed for flow cytometry analyses of cell cycle (lower right panel). Panel *P < 0.01 relative to DMSO-treated control at the same cell cycle phase based on *t* test. **b** Results of MTT assays and western blot analyses of HEXIM1 and GAPDH expression. *P < 0.01 relative to DMSO-treated control shRNA-transfected cells of the same cell line based on *t* test. **c** MDA-MB-231 cells were transfected and/or treated with KDOAM25 (500 nM) for 10 h as indicated and processed for Western blot analyses. Figures are representative of at least three experiments

The use of HEXIM1 inducers as differentiating factors to push cancerous cells towards quiescence would be advantageous as a therapeutic strategy in comparison with cytotoxic agents. HEXIM1 is required for HMBA- and 4a1-induced differentiation and upregulation of p21 expression [13], which is known to promote cellular differentiation [33]. We also examined the ability of KDOAM25 to induce differentiation. Nile red staining to detect lipid droplets (markers of differentiation) indicate that KDOAM25 also induced differentiation (Fig. 6a). KDOAM25 induced p21 expression and/or differentiation of TNBC cell lines tested that express mutant p53--BTB549, MDA-MB-231, and MDA-MB-468 (Fig. 6a). Consistent with KDOAM25-induced increase in p21 expression is the increase in fraction of cells in the G1 and G2 phases.

### Regulation of proliferation, differentiation, and certain growth regulatory factor by KDOAM25 requires HEXIM1

KDM5B has been proposed as a repressor of tumor suppressor genes via removal of the activating H3K4me3 marks [34]. While correlative changes in gene expression were observed as a result of alterations in KDM5B expression, primary mediators of the pro-proliferative action of KDM5B were not well-defined. We tested the possibility that while KDM5B and its inhibitors likely regulate other targets, HEXIM1 is a critical mediator of the anti-cancer effects of KDM5B inhibitors. Our data indicate that HEXIM1 is required for the induction of differentiation and growth inhibition via KDOAM25 (Fig. 6a, b). While KDOAM25 treatment resulted in decreased levels of Myc and Cyclin D1, this inhibitory action was attenuated upon downregulation of HEXIM1 with shRNA (Fig. 6c). Thus, HEXIM1 was required for these actions of KDOAM25.

### Modulation of the response to cancer chemotherapy by HEXIM1 inducers

**Fig. 7:**
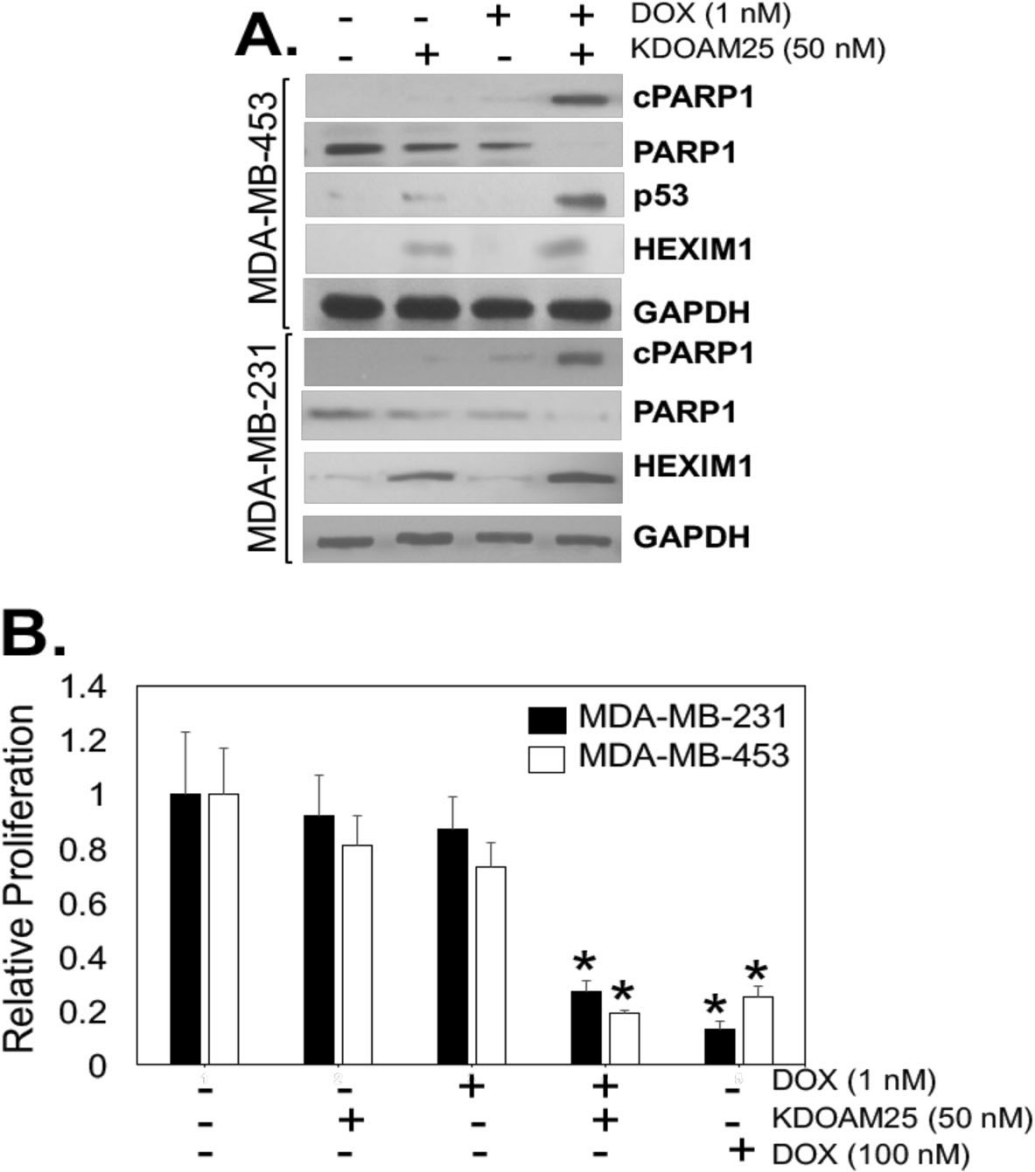
HEXIM1 and KDOAM25 induced increased sensitivity to doxorubicin. **a** TNBC lines were treated with vehicle, KDOAM25, and/or doxorubicin (at indicated concentrations) for 18 h and processed for western blot analyses of indicated proteins. **b** TNBC lines were treated with doxorubicin ± KDOAM25 for 6 days then processed for MTT assays to assess proliferation. *P < 0.01 relative to DMSO-treated cells of the same cell line based on *t* test. Figures are representative of at least three experiments

As reported above, higher HEXIM1 expression is also associated with longer survival of chemotherapy-treated patients (Fig. 1b). We determined whether induction of HEXIM1 expression would result in enhanced sensitivity to doxorubicin using a TNBC line, MDA-MB-453, which exhibited lower sensitivity to KDM5B inhibitors compared to other TNBC lines tested. We observed that suboptimal concentrations of KDOAM25 and doxorubicin synergized in inducing apoptosis and inhibiting cell growth (Fig. 7). KDOAM25 alone did not induce apoptosis, consistent with our FACS analyses showing a non-significant increase in apoptotic cells after treatment (Fig. 6a). However, KDOAM25-induced G2/M arrest may have resulted in genomic instability that “primes” cells for the induction of apoptosis by low levels of doxorubicin, which can occur in p53-independent manner [35]. G2/M arrest has been reported to result from treatment with certain cancer chemotherapy agent such as paclitaxel and result in polyploidy or multinucleation, followed by apoptosis [36–38].

### In vivo testing of KDOAM25

**Fig. 8:**
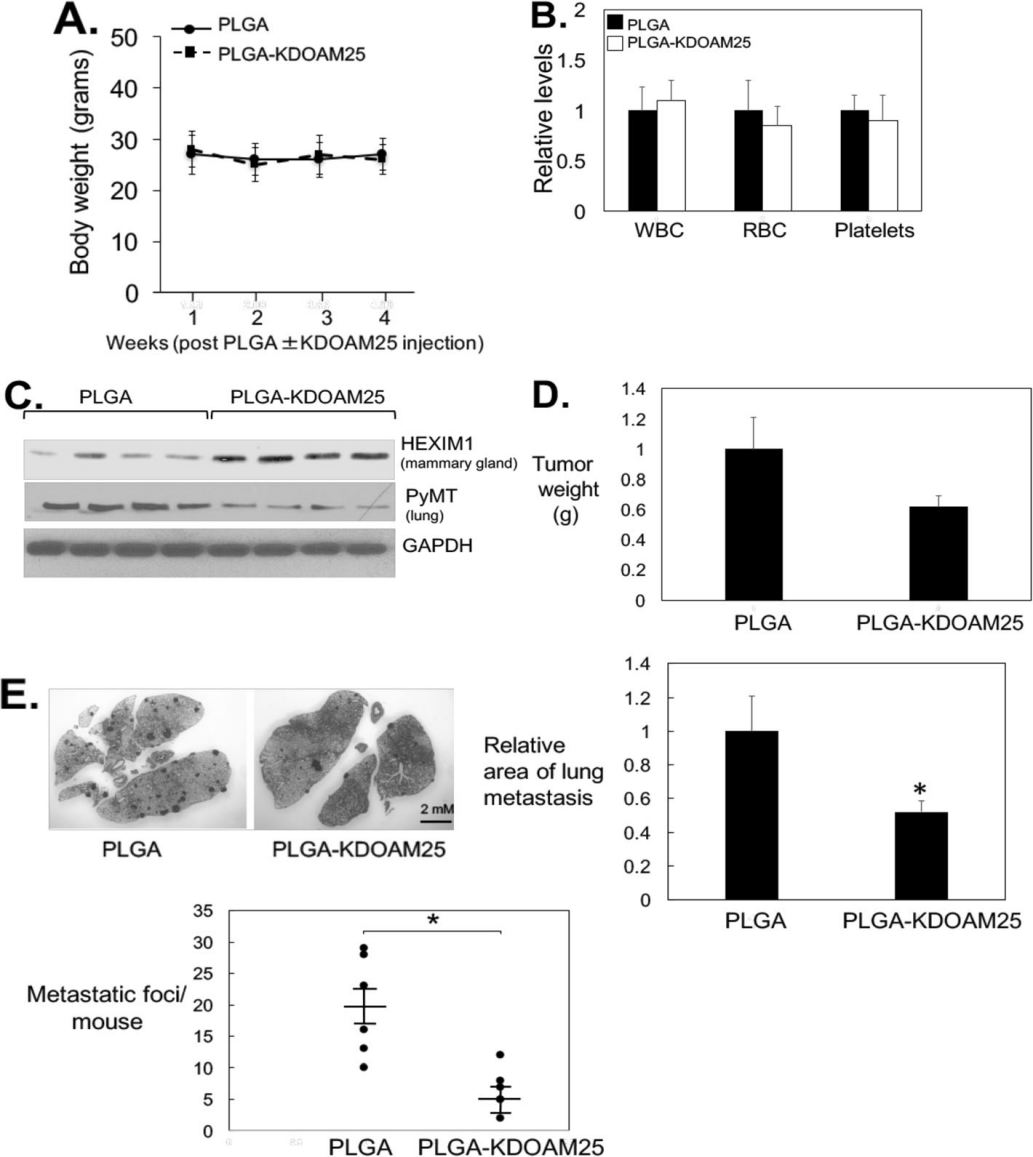
In vivo testing of KDOAM25 activity. PLGA or PLGA-KDOAM25 (1 μM, 50 μl volume) were injected into mammary glands once/week for 4 weeks. **a** Body weights were monitored weekly. **b** Blood cell levels were determined using the HEMAVET 950FS. **c** Western blot analyses of indicated proteins in the mammary gland. **d** Final tumor weights and **e** upper left panel: lungs from PLGA ± KDOAM25-treated PyMT mice. Upper right panel: quantification of tumor area in hematoxylin and eosin-stained lung tissues. Lower left panel: number of metastatic lesions visible in hematoxylin and eosin-stained lung tissues in the lungs of each mouse. Panels represent 5–6 mice per group (PLGA ± KDOAM25). *P < 0.05 relative to PLGA-treated mice based on *t* test

The Polyoma Middle-T antigen (PyMT) transgenic mouse is a well-characterized model of human metastatic breast cancer used for preclinical testing [39]. We utilized the PyMT mice to test the effectiveness of KDOAM25 in inhibiting tumor metastasis and used the same delivery system and treatment schedule as we used previously for 4a1 [13]. KDOAM25 is well tolerated as indicated by lack of significant weight loss and no decrease in platelet levels (absence of thrombocytopenia) (Fig. 8a, b). PLGA-mediated delivery of KDOAM25 induced increases in HEXIM1 expression in the mammary gland (Fig. 8c), as well as decreases in tumor weights, although the decrease was not statistically significant (Fig. 8d). Decreases in macrometastatic lesions were evident in the lung of KDOAM25-treated PYMT mice (Fig. 8e)**.** We took advantage of the mammary-specific expression of PyMT to detect individual early lesions or micrometastasis tumor cells in lung tissue and assessed PyMT expression in the lungs. We observed decreased PyMT levels in the lungs of PLGA-KDOM25-treated PyMT mice when compared to control mice (Fig. 8c).

## Discussion

We utilized 4a1, a more potent derivative of the differentiating agent HMBA, as a bait in a chemical proteomics approach to identify direct interacting targets. We identified a highly druggable target, KDM5B (JARID1B/PLU1), that interacts with HMBA and 4a1 to induce HEXIM1. Our data suggest that HMBA and 4a1 induce HEXIM1 expression by inhibiting KDM5B, resulting in an increase in activating H3K4me2/3 marks on the *HEXIM1* regulatory region. Consistent with our findings, known inhibitors of KDM5B were also able to induce HEXIM1 expression. While KDM5B and its inhibitors likely regulate other targets, our studies are the first to show that induction of HEXIM1 expression is required for ability of KDM5B inhibitors to (1) induce p21 expression and differentiation, (2) inhibit expression of oncogenic genes such as cyclin D1 and Myc, and (3) inhibit breast cancer cell proliferation. Identification of novel molecular scaffolds, such as 4a1, that more potently induced HEXIM1 expression and supporting data which show KDM5B as a target of these compounds, we opened up a new lead discovery and optimization directions. Our collaborator recently reported that HMBA/4a1 are putative activators of HSP70 [40]. However, HSP70 is likely a much lower affinity target for these compounds and a less desirable pharmaceutical target than KDM5B, given the broad functions of HSP70.

In recent years, KDM5 family members have been presented as highly druggable targets for the development of epigenetic modulators of various cancers [41–44]. Numerous groups have attempted to drug the JmjC catalytic core via the design of competitive inhibitors that are analogs of the essential 2-OG co-substrate [41–44]. While developing small molecule leads that are very potent in biochemical assays, these groups typically found that the 2-OG analogs performed poorly in intact cells, with potencies dropping from nanomolar in vitro to micromolar on cells. This is most likely due to direct competition with endogenous 2-OG that, as a key intermediate of the Krebs cycle, is highly abundant in the 100-μM range or above. This scenario is further exacerbated in cancerous cells that also produce the oncometabolite, 2-hydroxyglutarate (2-HG), which can accumulate inside cells above millimolar levels [45] and also binds to KDM5B [46]. Such effects are perhaps the best explanation of why TNBCs (which typically produce high levels of 2-HG) are among the most refractory breast cancer cell lines to treatment with most KDM5B inhibitors [28]. KDOAM25 works better on cells than most published KDM5B inhibitors, perhaps because the compound also extends into the methylated lysine histone binding region of JmjC and is able to partially occlude access of the substrate being presented for demethylation. Guided by biology rather than chemistry in developing chemical tools from HMBA, we identified alternative binding sites within the JmjC catalytic core that, when occupied by inhibitory ligand, probably occlude the demethylation substrate (i.e., H3K4me2/3) without competition from the abundant intracellular pools of 2-OG and/or 2-HG. In a collaboration between the Montano laboratory and OncoStatyx LLC, we have discovered additional proprietary KDM5B inhibitors with chemotypes unrelated to 4a, which induce HEXIM1 protein expression, induce p21-mediated quiescence, and inhibit proliferation of multiple TNBCs at low nanomolar potency/efficacy on cells (manuscript in preparation).

Differentiating agents are advantageous as therapeutic agents when compared to cytotoxic compounds due to the absence of effects on non-tumorigenic cells. Moreover, differentiation therapy aimed at favoring differentiation over programs of self-renewal induced a depletion of the CSC population within tumors [47]. Differentiation was assessed via the upregulation of lipid droplets and p21 expression. p21 promotes cellular differentiation, and loss of p21 in breast cancer cells results in the acquisition of stem cell properties. In agreement with this model, 4a1 downregulated Nanog expression and induced differentiation of the stem cell fraction in TNBC [13]. Our results indicate that HEXIM1 is required for the induction of differentiation of TNBC cells using KDOAM25. p53 is a major regulator of p21, and HEXIM1 enhances the stability of p53 [48]. However, p53 is highly mutated in cancers, and this has hampered the clinical use of differentiating agents. KDOAM25 treatment induced p21 expression and/or differentiation of TNBC cell lines that express mutant p53, suggesting a p53-independent induction of p21 and differentiation of TNBC cells as a result of HEXIM1 induction.

We have previously reported that HEXIM1 inhibits metastasis by inhibiting cell invasion, angiogenesis, and the premetastatic niche [6, 7, 49, 50], partly via direct downregulation of HIF-1α and VEGF expression [7].) An alternative mechanism for inhibition of metastasis by HEXIM1 inducers involves the induction of differentiation. Increasing evidence suggests that a subpopulation of cancer stem cells (CSCs) contributes to metastasis. Breast cancer stem cells (BCSCs) are not only a consequence of enhanced self-renewal ability of normal stem cells but also a result of the de-differentiation of cancer cells. Thus, the downregulation of the CSC population by HEXIM1 inducers as we have previously reported [13] may contribute to inhibition of metastasis by these agents.

In determining that HMBA and 4a1 induce HEXIM1 expression by counteracting the inhibitory effects of KDM5B upon HEXIM1 expression, we may have perhaps determined how HEXIM1 expression is lost in breast cancer. KDM5B is overexpressed in breast cancer and thus likely inversely correlated with HEXIM1 expression. Of note, search of COSMIC and TCGA databases indicates that KDM5B and HEXIM1 mutations are not common occurrences in breast and prostate cancer. Thus, the altered expression of KDM5B and HEXIM1 in tumors, rather than mutations per se, are the likely driving factors in breast tumorigenesis. Paradoxically, HEXIM1 upregulated KDM5B expression in prostate cancer [8]. However, KDM5B expression is not upregulated by HEXIM1 in TNBC, suggesting cell-specific regulation of KDM5B expression by HEXIM1.

Current oncology practice is moving towards the use of multiple targeted therapeutics due to the significant problem of cancer cells developing resistance via adaptive or mutational bypass to therapeutic agents which target singular pathways [51, 52]. HEXIM1 inhibits tumor growth and metastasis by inducing cancer cell differentiation and inhibiting cell proliferation and invasion, angiogenesis, and the premetastatic niche [6, 7, 49, 50]. The simultaneous targeting of multiple pro-cancer pathways by HEXIM1 improves the likelihood of sustained effect by limiting the cancer cell’s ability to bypass the inhibition of any one pathway. Moreover, the ability of HEXIM1 to inhibit major tumor promoting pathways likely contributes to the effective targeting of multiple breast cancer subtypes with HEXIM1 inducers, alone or in combination with other therapeutics.

## Conclusion

HMBA and 4a1 induce HEXIM1 expression by inhibiting KDM5B, a result that was replicated with known KDM5B inhibitors. Upregulation of HEXIM1 expression levels plays an essential role in the inhibition of proliferation of breast cancer cells via KDM5B inhibitors. Based on the novel molecular scaffolds that we identified which induced HEXIM1 expression more potently and data in support that KDM5B is a target of these compounds, we have opened up new lead discovery and optimization directions.

## Supplementary information

**Additional file 1.** Supplementary methods

## Data Availability

All data are available within the article. All materials are available from the authors upon request.

## Abbreviations

2-OG: 2-Oxoglutarate/α ketoglutarate
ChIP: Chromatin immunoprecipitation assay
ERα: Estrogen receptor alpha
GAPDH: Glyceraldehyde-3-phosphate dehydrogenase
H3K4me2/3: Histone 3 dimethylated/trimethylated at lysine 4
HEXIM1: Hexamethylene-bis-acetamide (HMBA)-inducible protein 1
KDM5B: Lysine (K)-specific demethylase 5B
PBIT: 2–4(4-Methylphenyl)-1,2-benzisothiazol-3(2H)-one
PLGA: Poly (lactic-*co*-glycolic acid)
PyMT: Polyoma Middle-T antigen
RT-PCR: Real-time polymerase chain reaction
SDS-PAGE: Sodium dodecyl sulfate polyacrylamide gel electrophoresis
shRNA: Small hairpin ribonucleic acid
TNBC: Triple-negative breast cancer
