# Supplementary material for: Inhibition of the histone demethylase, KDM5B, directly induces re-expression of tumor suppressor protein HEXIM1 in cancer cells
Source: Breast Cancer Res. 2019 Dec 5;21:138. doi: 10.1186/s13058-019-1228-7 (PMC6896798; doi:10.1186/s13058-019-1228-7)
Supplement: Supplementary file 1 — Additional file 1. Supplementary methods [file 13058_2019_1228_MOESM1_ESM.docx]

**SUPPLEMENTARY METHODS**

**Biotin-NeutrAvidin Pull-Down Assay.** MDA-MB-231 cells (10^7^) were disrupted in NP-40 lysis buffer with protease inhibitor cocktail and sonicated. The lysate was incubated with biotin-conjugated 4a1 (200 μM) at room temperature for 1h. In some experiments the mixture was further incubated with equilibrated NeutrAvidin resin in columns at room temperature for 30 min, followed by centrifugation and five washes with binding buffer to wash out non-specific binding. Proteins bound to the biotinylated 4a1 were eluted using binding buffer containing 4a1 (200 μM), HMBA (1 mM), or 3e2 (1 mM, a structurally related but inactive HMBA analog, ref.(14)). The eluate was boiled with loading buffer (100 mM DTT plus bromophenol blue) for 5 minutes and then electrophoresed on a 10% SDS-polyacrylamide gel. The resulting gel was visualized with coomassie blue staining (for Mass Spectrometry). In some experiments, proteins resolved in the gel were transferred onto nitrocellulose filters for western blot analyses.

**Mass Spectrometry.** Bands visualized by coomassie blue staining and a total of 12 gel bands were in-gel digested using 150 ng of trypsin per each gel band. LC-MS analysis of all digested protein bands were carried out on the Orbitrap Elite mass spectrometer (Thermo Electron, San Jose, CA) interfaced with a Waters nanoAcquity UPLC system (Waters, Taunton, MA). Proteolytic peptides from each gel band were desalted on a trap column (180 μm × 20 mm packed with C18 Symmetry, 5 μm, 100 Å (Waters, Taunton, MA)) and subsequently eluted on a reverse phase column (75 μm x 250 mm nano column, packed with C18 BEH130, 1.7 μm, 130 Å (Waters, Taunton, MA)) using a gradient of 2 to 45% mobile phase B (0.1% formic acid and acetonitrile (ACN)) vs. mobile phase A (100% water/0.1 % formic acid) over a period of 60 minutes at 37 °C with a flow rate of 300 nl/min. Peptides eluting from the column were introduced into the nano-electrospray source at a capillary voltage of 2.5 kV. For MS analysis, a full scan was recorded for eluted peptides (*m/z* range of 350–1800) in the FT mass analyzer with resolution of 120,000 followed by MS/MS of the 20 most intense peptide ions scanned in the ion trap (IT) mass analyzer. All MS data were acquired in the positive ion mode. The resulting MS/MS data were initially searched against human proteins database using Mascot software engine to identify proteins from each gel band. In particular, MS/MS spectra were searched for tryptic peptides from human protein sequences derived from IPI database using mass accuracy values of 8 ppm and 0.7 Daltons for MS and MS/MS scans respectively, with allowed variable modifications including carbamidomethylation for cysteines and oxidative modification for methionine amino acid. To call a protein identified, we further analyzed all search files derived from Mascot analysis using Scaffold software with the following criteria in Scaffold: peptide identification probability 95%; protein identification probability 99%, and at least two peptides per protein. These proteins were subsequently quantified by Scaffold using normalized spectral counting algorithm; protein fold change of more than 3-fold (for all proteins from control and treated samples) was considered significant.

**Purification of KDM5B JmjC domain.** KDM5B cDNA cloned into pFB-LIC-Bse (from Structural Genomics Consortium, University of Oxford, UK) was expressed in Sf9 cells using the Baculovirus Expression Vector System following manufacture’s protocol (Invitrogen, Waltham, MA). Briefly, the recombinant bacmid was isolated from *E. coli* strain DH10Bac and transfected into insect cells using Cellfectin II Reagent. The KDM5B expressing baculovirus was amplified in Sf9 insect cells grown in ESF 921 media from Expression Systems (Davis, California). For protein production, Sf9 cells were transduced with baculovirus and grown in the same media. Pellets of Sf9 cells producing KDM5B were frozen at -80 °C until protein processing.

For protein purification, pellets were resuspended in lysis buffer containing 20 mM HEPES, 100 mM NaCl, 10 mM imidazole, 10 mM β-mercaptoethanol, 10% Glycerol, pH 7.5. Cells were lysed in a microfluidizer. Membranes and insoluble material were removed by centrifugation at 100,000 *g* for 60 min. The supernatant containing KDM5B was purified with immobilized metal affinity chromatography (IMAC) using Ni-NTA beads (Invitrogen). KDM5B was eluted from the column with 250 mM imidazole. Further purification was done with FPLC using a Superdex 200 column and buffer was exchanged to 20 mM HEPES, 100 mM NaCl, 0.05% Tween 20, 1 mM DTT, pH 7.5. The protein was flash frozen in liquid nitrogen and stored at -80 °C. The purified KDM5B Jmj domain was used in Surface Plasmon Resonance studies or in biotin-NeutrAvidin pull-down assays to quantify or validate interaction with biotin-conjugated 4a1 or HMBA, respectively.

**Surface Plasmon Resonance.** SPR studies were performed using a Biacore T100 (GE Healthcare, USA) in 25 mM HEPES (pH 7.5), 100 mM NaCl. KDM5B Jmj domain was covalently coupled to S series CM5 sensor chips via amine coupling to a density of approximately 8000RU. Series of concentrations of drug 4a1 were injected at 30 mL/min over the surface where protein was immobilized. Each concentration was repeated twice. All sensorgram traces had first subtracted the reference response and then subtracted the zero-concentration. Data were analyzed using BIAEvaluation software (GE Healthcare) with steady state fitting model.

**Docking of HEXIM1 inducers onto KDM5B.** Coordinates for the KDM5B-KDOAM25 complex were retrieved from the PDB (accession code 5A3N). Water molecules and alternative side chain conformations were removed from the file using the program PDBSET from the CCP4i software suite. The active site non-native Mn atom was replaced with Fe. Coordinates for a KDM4D in complex with 2-OG were retrieved from the PDB (accession code 4HON) and superimposed on the KDM5B coordinates. Residues contacting the 2-OG molecule are well conserved between the two structures. The 2-OG coordinates were then added to those of the KDM5B molecule and side chains surrounding the ligand were adjusted appropriately based on the KDM4D molecule. This KDM5B-2-OG model was subjected to energy minimization in the program REFMAC. The resulting file was then converted into .pdbqt format required for docking calculations using AutoDock tools. Coordinate files for 4A1 and hexamethylene-bis-acetamide (HMBA) were generated using the GRADE server and converted to .pdbqt format using Autodock tools. Single bonds were treated as freely rotatable. Docking calculations were carried out using Autodock Vina. The docking search space was restricted to a 25 Å region surrounding the non-heme active site Fe. The exhaustiveness parameter was set to 160 for both ligand docking calculations. The results of the docking calculations were rendered using PyMOL.

**Antibodies**. Antibodies used for chromatin immunoprecipitation and immunoblotting include: HEXIM1 (generated in Montano laboratory, (6), KDM5B (Bethyl Laboratories, Montgomery, TX, USA), PyMT (Calbiochem, San Diego, CA, USA). Antibodies against the following were obtained from Cell Signaling Technologies (Danvers, MA, USA): cyclin D1, myc, cleaved PARP1, p53. Antibodies against the following were obtained from Santa Cruz Biotechnology (Dallas, TX, USA): p21 and PARP1. Antibodies against the following were obtained from Millipore (Temecula, CA, USA): H3K4me2, H3K4me3, and GAPDH.

**Generation of shRNA lentiviruses and transduction into cells.** MISSION shRNA plasmids used to generate lentiviruses were obtained from Sigma-Aldrich (St. Louis, MO). To generate lentiviral particles, 293FT cells (Invitrogen) were transfected with envelope expressing plasmid (pMD2.G), packaging plasmid (psPAX2) and shRNA expression plasmid (pLKO) using Lipofectamine 2000 (Invitrogen). Media containing lentiviral particles were collected 40 to 68 h post-transfection and filtered with 0.45 μm filters prior to storage at -80 C. Breast cells were transduced with lentiviruses in the presence of 10 μg/ml polybrene. After overnight incubation in the CO_2_ incubator, cells were treated with puromycin (250 ng/ml) to select for transduced cells for 36 hours.

**Proliferation assay.** Cells were plated onto a 96-well plate at a density of 3000 cells/well. Some cells were infected with control shRNA or HEXIM1 shRNA lentiviruses for 48 hours prior to plating for proliferation assays. Cells plated onto 96-well plate were fed with fresh media containing DMSO, 4a1, KDOAM25 and/or Doxorubicin every other day. After 6 days, cell proliferation was assessed using the MTT based Cell Growth Determination Kit (Sigma-Aldrich). Absorbance was measured at 570 nm using a Molecular Devices plate reader (San Jose, CA)

**Lipid Droplets (Nile Red Staining).** Cells were plated onto the wells of a 24-well plate containing coverslips at a density of 3x10^4^ cells/well. Some cells were infected with control shRNA or HEXIM1 shRNA lentiviruses for 48 hours prior to plating for proliferation assays. Twenty four hours after plating, cells were treated with DMSO or KDOAM25. After 24 hours, cells were stained with Nile red for lipid droplets (marker of cell differentiation). Briefly, the stock solution of Nile red 1 mg/ml in acetone was diluted in PBS (1:1000). The fixed cells (4% paraformaldehyde) were incubated with diluted Nile red for 5 minutes at room temperature, rinsed with PBS and observed for the presence of lipid droplets by confocal microscopy. Images were digitally captured using a Leica microscope (Leica Microsystems Inc, IL, USA). The intensity of Nile red staining and the number stained cells relative to the total number of cells were quantified from five different fields of vision. The product of the two values from KDOAM25-treated cells were normalized to the product of the two values from DMSO treated cells.

**Chromatin Immunoprecipitation.** Cells were grown in 100-mm dishes. Some cells were infected with control shRNA or KDM5B shRNA lentiviruses and/or treated with DMSO or KDOAM25 48 hours or 2-5 hours prior to processing for ChIP assays, respectively . ChIP analyses have been described previously (6). Briefly, cells were fixed with 1% formaldehyde and lysed in SDS-lysis buffer with protease inhibitors. Lysed cells were sonicated using a Branson 450 sonicator with a 3-mm tapered microtip at power setting 3 and 70% duty for 10 pulses/cycle and nine cycles (
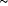
5-W output for 8 to 10 seconds). Clarified, sonicated chromatin was diluted 10-fold in chromatin immunoprecipitation (ChIP) dilution buffer. One milliliter of the diluted chromatin was used for overnight immunoprecipitation with a given antibody. The antibody-chromatin complexes were pulled down using protein A beads. The beads were subjected to a series of washes as described and the antigen-DNA complexes eluted. The eluates were reverse cross linked overnight at 65^0^ C and the DNA was purified by phenol: chloroform extraction. Ethanol precipitated pellets were resuspended in 50 μl of water and 2 to 4 μl of the suspension was used as template for PCR analysis. The reaction mix contained 10 mM Tris-HCl, 1.5 mM MgCl_2_, 50 mM KCl, 200 uM dNTP, 1 unit Tag DNA Polymerase for varying cycle numbers using 1 uM primers in a Corning PCR tube (6531) and Biometra Thermocycler. PCR-amplified products were run on a 2% agarose gel and visualized by ethidium bromide staining. The fluorescence was captured using the LI-COR Odyssey System, and quantified using the Image J software. Primers sequences are as follows:

hHEX1 -3179 fw: CTGTGATAAGGCTGGACTGC

hHEX1 -2595 rv: GCAACCTTACTGTTAGCTGGG

hHEX1 -920 fw:GAGGTCTAAGCGATGGAAGGT

hHEX1 -389 rv: CGAAGGGGTTAAAACTACCGA

**RT-PCR.** Cells were grown in 6-well plates. Four hours after treatment with DMSO or KDOAM24, cells were harvested and total mRNAs were extracted using TRIzol® reagent (Invitrogen) according to the manufacturer’s protocol. mRNAs were reverse transcribed using the M-MLV Reverse Transcriptase kit (Invitrogen) following the recommended protocol. One micro liter of the cDNA was PCR-amplified in reaction mix containing 10 mM Tris-HCl, 1.5 mM MgCl_2_, 50 mM KCl, 200 uM dNTP, 1 unit Tag DNA Polymerase for varying cycle numbers using 1 uM primers (listed below) in a Corning PCR tube (6531) and Biometra Thermocycler. The amplified products were run on a 2% agarose gel and visualized by ethidium bromide staining. Fluorescence was captured using the LI-COR Odyssey System, and quantified using the Image J software. Signals in each case were normalized to their respective *GAPDH* values to calculate the relative expression levels. Primer sequences are as follows:

hHEX1 fw: ATGGCCGAGCCATTCTTGTCAG

hHEX1 rv: GTACGGTTTCCAATGCCGCTT

hGAPDH fw: GTCATCATCTCTGCCCCCTCTGCT

hGAPDH rv: CTTCTTGATGTCATCATATTTG

**Western Blotting.** Cells were grown in 6-well plates. Ten hours after treatment with DMSO or KDOAM25, cells were harvested and processed for Western blotting as described previously (13). Whole cell lysates were prepared using mammalian protein extraction reagent (M-PER) from Thermo Scientific. Fifty μg of the total protein extract was separated on a 8-10% SDS-polyacrylamide gel and electrophoretically transferred onto nitrocellulose membrane (Pall Corporation, Pensacola, FL). Membranes were blocked with 5% BSA and probed with the indicated primary antibodies overnight. The housekeeping gene, glyceraldehyde-3-phosphate dehydrogenase (GAPDH), was used as a loading control. The membranes were probed with HRP-conjugated anti-rabbit IgG or anti-mouse IgG secondary antibodies (1:5000). Signals were detected using the ECL Western Blotting Analysis System (GE Healthcare) or SuperSignal West Femto Chemiluminescent Substrate kit (Thermo Fisher Scientific). Signals were visualized using the LI-COR Odyssey System, and quantified using the Image J software. Signals in each case were normalized to their respective GAPDH values to calculate the relative expression levels.

**Kaplan–Meier (KM) analyses.** The Kaplan-Meier plotter (<http://kmplot.com/analysis/)> or Breast Cancer Gene-Expression Miner Version 4 (http://bcgenex.centregauducheau.fr/BC-GEM/GEM-Accueil.php?js=1) were used to correlate mRNA levels of *HEXIM1* or *KDM5B* to patient outcome in breast tumor datasets. For relapse-free survival (PFS) analysis, all results were used for auto select best cutoff and exclude biased arrays

**Mouse models.** All mice in this study were generated using FVB mouse strain. In order to examine the effects of KDOAM25 on tumorigenesis and metastasis, we utilized the PyMT mice. The PyMT transgene was detected using the primers: PyMT fwd: gga AgC AAg TAC TTC ACA Agg g, PYMT rev: ggA AAg TCA CTA ggA gCA ggg. Tumor latency was determined as the first day a tumor was palpated, typically around 8 weeks of age. Fifty ul of PLGA or PLGA-KDOAM25 were then injected into the left thoracic mammary glands of PyMT mice, every other week. Control PLGA-treated and PLGA-KDOAM25-treated PyMT mice were from the same litter. Tumor volume and body weights were assessed weekly. To allow for detection of metastasis to the lung, PyMT mice that were treated with PLGA or PLGA-KDOAM25 were sacrificed at 15 weeks of age. The left thoracic mammary tumors were collected and divided into 2 parts for western blots or paraffin sections. Lungs were also collected and processed as described below to assess metastasis. Blood were collected to assess blood cell levels using the HEMAVET 950FS.

**Immunohistochemistry.** Mice lungs were fixed in 10% formalin, embedded in paraffin, and sectioned (10 μm) for immunohistochemistry experiments. H&E-stained lungs were quantified using Metamorph offline program version 7.6 (MDS analytical technologies). The areas infiltrated by tumors and the total area of the lungs were calculated by setting different color thresholds between lesions and normal lung tissues. The ratio of the areas infiltrated by tumors and total area of the lungs was calculated as relative area of lung metastasis.
